# A prospective clinical study of polycarboxylate cement in periapical surgery

**DOI:** 10.4317/medoral.17457

**Published:** 2011-12-06

**Authors:** María Peñarrocha-Diago, Bárbara Ortega-Sánchez, Berta García-Mira, Laura Maestre-Ferrín, David Peñarrocha-Oltra, Cosme Gay-Escoda

**Affiliations:** 1 Associate Professor of Oral Surgery. Valencia University Medical and Dental School; 2 DDS. Master in Oral Surgery and Implantology. Valencia University Medical and Dental School; 3 DDS. Student of Master of Oral Surgery and Implantology. Valencia University Medical and Dental School; 4 Chairman of Oral Surgery. Director of the Master in Oral Surgery and Implantology. Barcelona University Medical and Dental School (Spain)

## Abstract

Objective: To evaluate the clinical efficacy of polycarboxylate cement as retrograde filling material. Design: A prospective clinical study was made of 25 patients subjected to periapical surgery with ultrasound and magnifying loupes, in which polycarboxylate cement was used as retrograde filling material. Measurements were made of the area and diameter of the lesions pre- and postoperatively, and 6 and 12 months after the operation. The apical resection and retrograde filling areas were also measured, and the prognosis following surgery was recorded. Results: A total of 23 patients with 31 apicoectomized teeth were studied (2 patients being lost to follow-up). The mean area of the periapical lesions before surgery was 52.25 mm2, with a mean major diameter of 6.1 mm and a mean lesser diameter of 4.8 mm. The success rate after 12 months was 54.7%, according to the criteria of Von Arx and Kurt. The prognosis was poorer in females, in larger lesions, and in cases with larger retrograde filling areas. Conclusions: Polycarboxylate cement offers good results, with important bone regeneration after periapical surgery.

** Key words:** Periapical surgery, endodontic treatment, polycarboxylate cement.

## Introduction

Polycarboxylate cement was developed by Smith in 1968 (1). Its main advantage is strong adhesion to dentin (1). This cement is composed of an aqueous solution of polyacrylic acid and inorganic salts, with zinc oxide as the main ingredient. Zinc is an essen-tial element, since it is needed for cell growth and differentiation (2); however, it also exhibits relative toxicity related to its ab-sorption and excretion (3).

 Nevertheless, in vitro studies (4) have demonstrated the sealing capacity and biocompatibility of polycarboxylate cement. In addition, following calcium hydroxide, it is the cement which preserves the largest presence of odontoblasts in the vicinity of the restorations (5) – thus justifying its use in periapical surgery (6).

The present study evaluates the outcome of periapical surgery with ultrasound, using polycarboxylate cement as retrograde filling material.

## Material and Methods

 Study sample

A prospective clinical study was made between January and December 2004, involving 25 patients subjected to periapical surgery with the ultrasound technique and using magnifying loupes to prepare the retrograde filling cavities. Polycarboxylate cement was used as retrograde filling material.

The following inclusion criteria were established: 1) apicoectomized teeth with canals subjected to ultrasound treatment for preparation of the cavities; 2) at least 12 months of follow-up after the intervention; and 3) retrograde filling with polycarboxylate cement.

 Surgical technique

**Figure 1:**
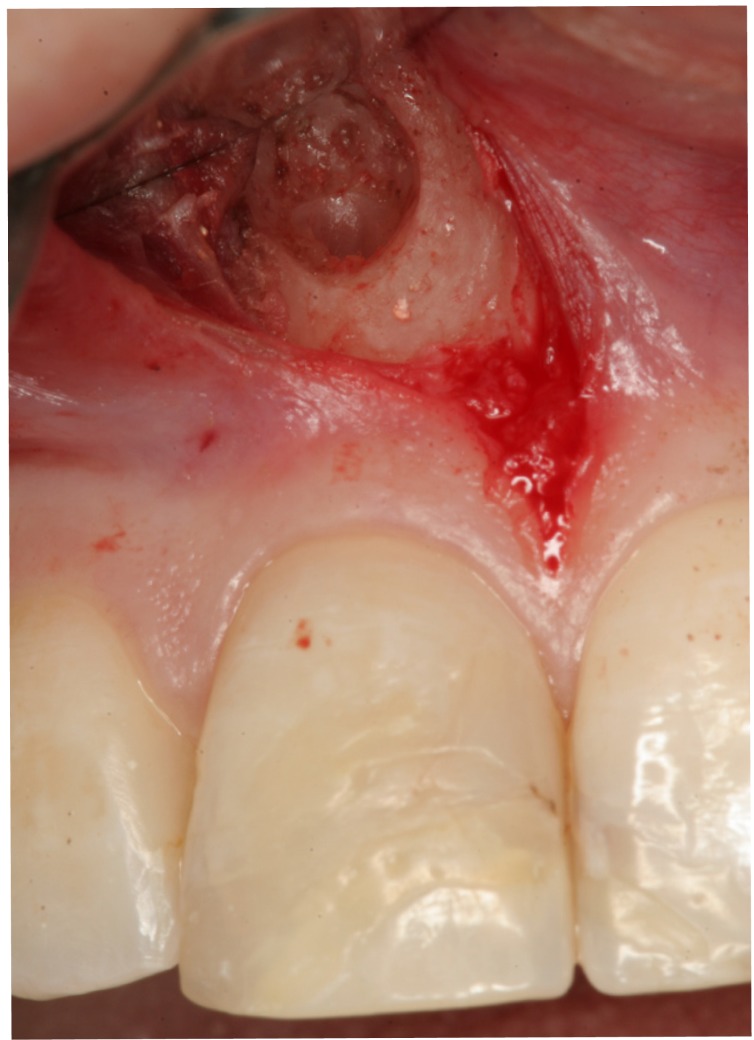
Retrograde filling cavity prepared with ultrasound tips.

**Figure 2:**
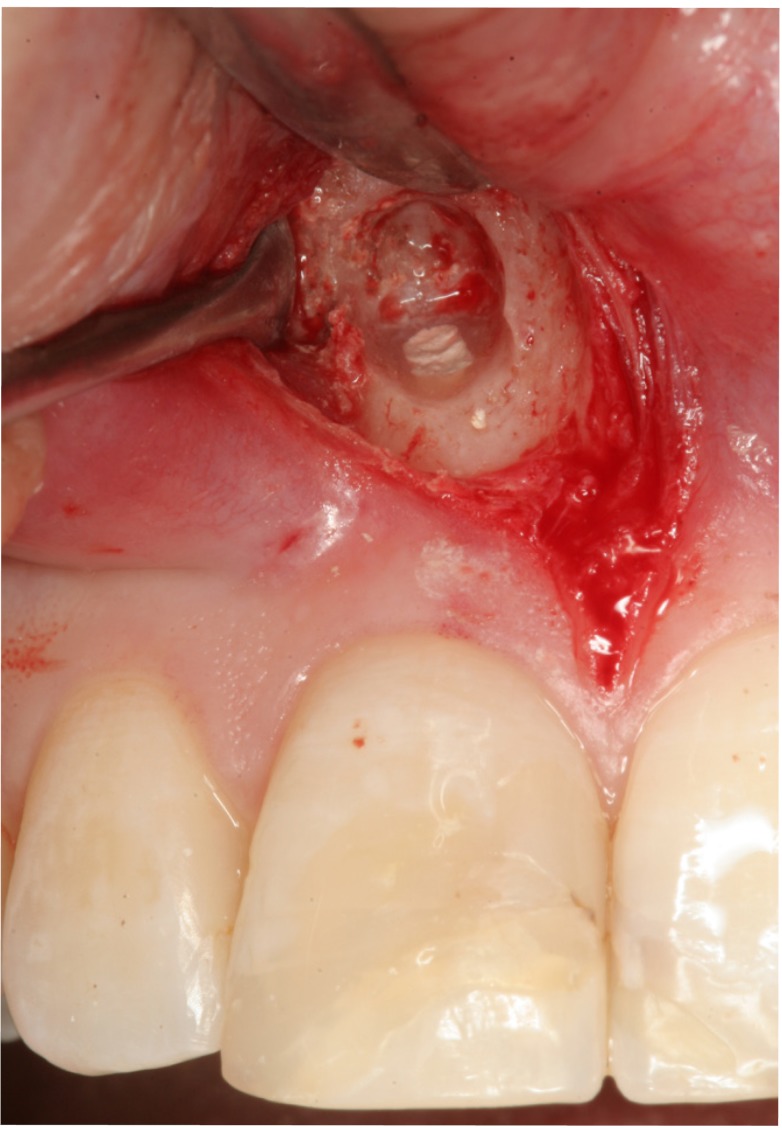
Retrograde filling with polycarboxylate cement.

All operations were carried out by the same surgeon (MPD). Locoregional and infiltrating anesthesia was used with 4% articaine and adrenalin 1:100,000 (Inibsa, Lliça de Vall, Barcelona, Spain). Full thickness Newman flaps (trapezoidal or triangular) were raised and ostectomy was carried out using a 0.27 mm round tungsten carbide drill (Jota, Switzerland) with abundant sterile saline irrigation. The minimal apical resection needed to gain access to the apex was performed, followed by curettage of the apical disease. The cavity was prepared for retrograde filling (Fig. 1) using a Piezon Master® ultrasound device (EMS, Electro Medical Systems S.A., Switzerland). To facilitate visualization of the root apexes, Orascoptic® loupes (magnification x 2.6) were em-ployed. Lastly, the polycarboxylate cement filler material was prepared, inserted and condensed (Durelon®, 3M Espe, USA) (Fig. 2), following the instructions of the manufacturer. Suturing was carried out with 4/0 silk thread (Lorca Marin®, TB15,3/8, Murcia, Spain).

 Radiographic evaluation

**Figure 3:**
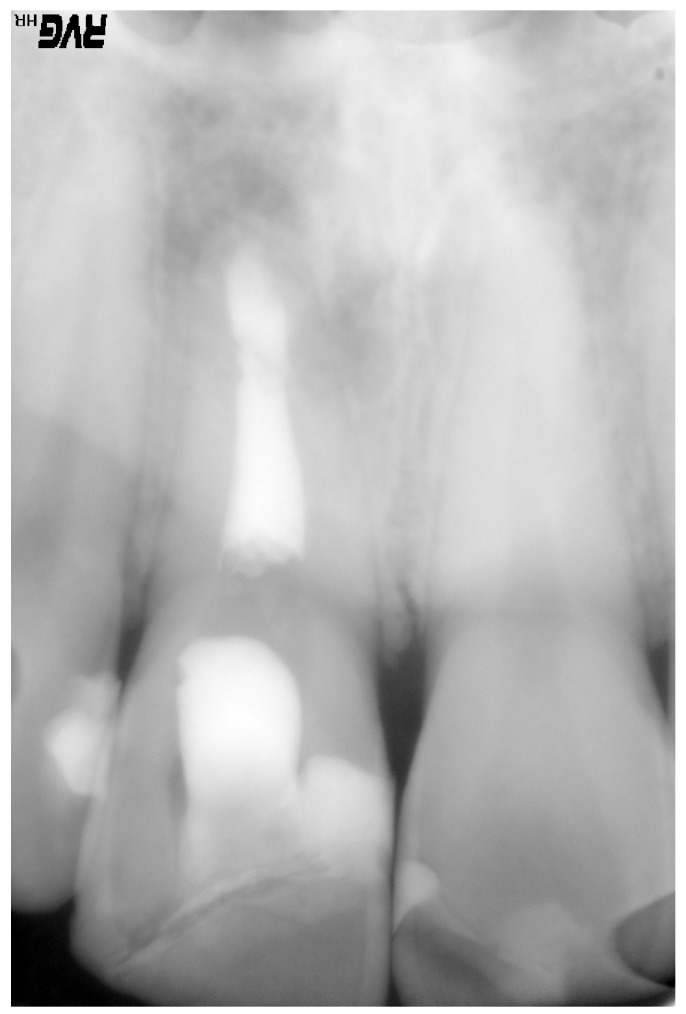
Postoperative control following periapical surgery.

Panoramic X-rays were obtained using a digital OP100® (Instrumentarium). An image analyzer was employed, with prior calibra-tion using the CliniView version 5.1 program.MicroImage Pro-Plus® (MediaCybernetics, Inc., Silver Springs, USA) was used to quantify the area (mm2) and the greater and lesser diameter (mm) of the lesion; the radiographic size of the lesion was evaluated before and immediately after the operation, and again 6 and 12 months after the intervention, on occasion of the last patient follow-up visits. In the postoperative X-ray study (Fig. 3) we determined the area (mm2), height (mm) and base (mm) of the apical resection, as well as the retrograde filling values.

The clinical and radiological findings were likewise documented 6 and 12 months after the operation, according to the criteria of von Arx and Kurt (1999) (7).

 Data collection and analysis

A coded protocol was applied in all patients, with an ordered and detailed registry of the clinical and radiographic data. The latter in turn were processed with the SPSS version 15 statistical package for Microsoft Windows. The associations between qualitative variables were examined with the chi-squared test, while quantitative variables were correlated using the Pearson coefficient. The pertinent mathematical assumptions were checked in all cases. Statistical significance was accepted for p<0.05.

## Results

A total of 23 patients were finally included (9 males and 14 females), with a mean age of 38.1 years (range 20-59), since two patients were excluded from the analysis due to a lack of follow-up. A total of 31 teeth (20 maxillary and 11 mandibular) were apicoectomized, with the filling of 53 roots and 61 canals. The mean duration of follow-up was 16.2 months (range 12-19). In terms of gender, statistically significant differences (χ2=7.442, p= 0.024) were observed regarding the overall outcome after 6 months, with a higher treatment success rate in males (60%) than in females (40%). 

Associated radiographic transparencies were observed in 80.6% of the patients, with a mean area of 52.25 mm2, a mean major diameter of 6.1 mm, and a mean lesser diameter of 4.8 mm. The data relating to the size of the apical resection and retrograde filling are reported in ( Table 1).

The outcomes after 6 and 12 months are shown in ( Table 2). The overall treatment success rate after 12 months was 54.7% (7). After this period of time one tooth was seen to have failed as a result of fracture. The statistically significant data are shown in ( Table 3), along with the differences after 6 and 12 months of follow-up. The larger the resection area, the poorer the outcome after 12 months. Likewise, the greater the obturation area, the poorer the outcome after this same period of time.

**Table 1:**
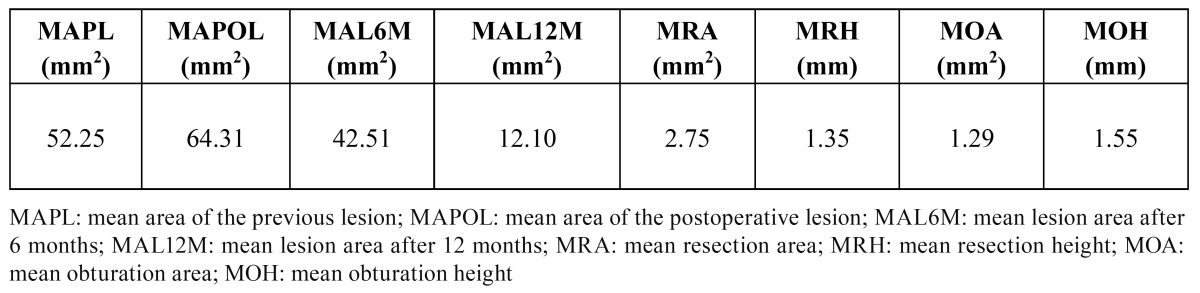
Dimensions del periapical area, apical resection and retrograde filling.

**Table 2:**
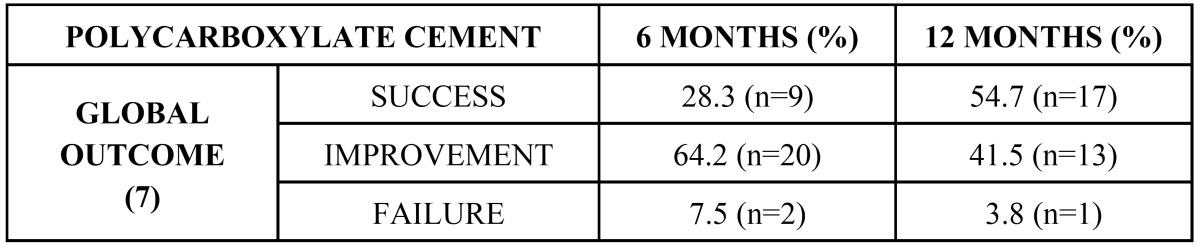
Treatment outcome with polycarboxylate cement.

**Table 3:**
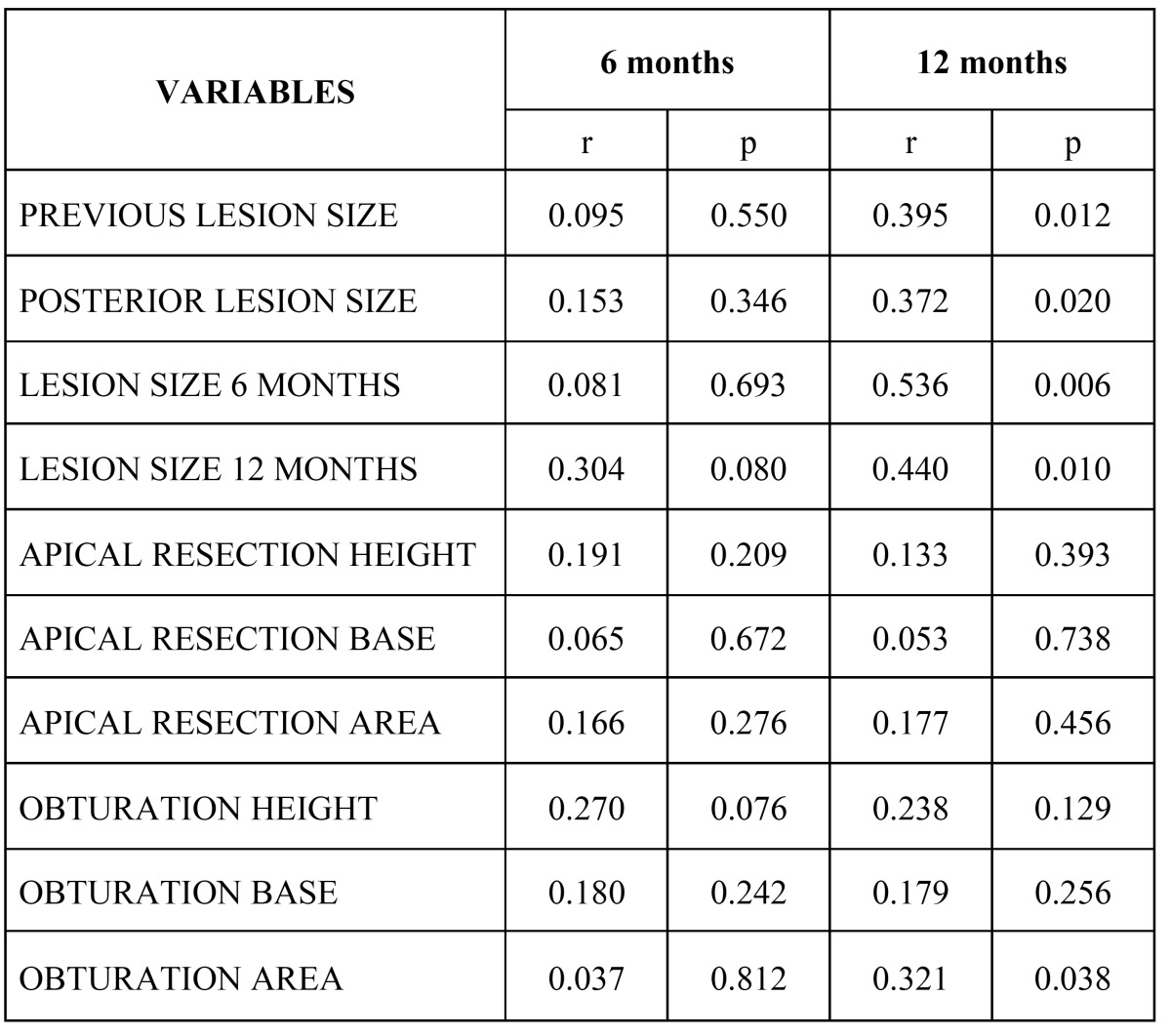
Outcome in relation to the studied lesion, apical resection and retrograde filling parameters.

## Discussion

Most publications on polycarboxylate cement in periapical surgery correspond to in vitro studies that examine properties such as the sealing efficacy of the material (8). In 1975, Barry et al. (4) compared the sealing capacity of silver amalgam, guttapercha and carboxylate cement (Durelon®) in extracted teeth. No significant differences were observed between guttapercha and silver amalgam, though Durelon® was seen to afford comparatively poorer sealing than the other two materials. In 1976, Barry et al. (9) examined the penetration of different dyes in cavities obturated with silver amalgam and with three different types of polycarboxylate cement (Durelon®, PCA and Poly C). All three cements were seen to present less dye penetration than silver amalgam. In 1993, Alhadainy et al. (10) compared the sealing capacity of different materials and found glass ionomer to afford the best sealing effect, followed by silver amalgam, guttapercha and polycarboxylate cement. Gargallo et al. (11) conducted a histological study in an animal model, comparing compomer and amalgam as filler materials. Comparatively more inflammation and expulsion of filler material beyond the root limits were recorded with compomer.

Regarding other studies that have used magnification loupes, Taschieri et al. (12), following the criteria of Molven et al. (13), compared the results of periapical surgery with magnification loupes versus endoscopy in 71 teeth filled with EBA®. The success rate in the endoscopy group was found to be 94% - with results similar to those of our own study.

Polycarboxylate cement offers good results, with important bone regeneration after periapical surgery, and is one of the cements that preserve the largest presence of odontoblasts in the vicinity of the restorations (5).
